# Clinical characteristics and management of primary granulocytic sarcoma of the oral cavity: A case report and literature review

**DOI:** 10.1097/MD.0000000000022820

**Published:** 2020-10-23

**Authors:** Yun-Gang Hu, Xiao-Hua Deng, Wei Lei, Xiao-Lin Li

**Affiliations:** a Department of Plastic and Maxillofacial Surgery, The People^'^s Affiliated Hospital of Nanchang University; b Key Laboratory of Maxillofacial Plastic and Reconstructive surgery, Jiangxi, People's Republic of China, 92 Aiguo road, Nanchang, Jiangxi, People's Republic of China.

**Keywords:** primary granulocytic sarcoma, oral tumor, clinical characteristics

## Abstract

**Introduction:**

Granulocytic sarcoma (GS) is a commonly occurring tumor comprising immature myeloid cells, which are usually related to acute or chronic myelocytic leukemia. The tumor rarely precedes leukemia without bone marrow involvement and is called primary GS. Although primary GS can occur in any body part, the involvement of the oral cavity is uncommon.

**Patient concerns:**

A 49-year-old woman hospitalized at the Department of Plastic and Maxillofacial Surgery presented with a growing mass in her left maxillary hard palate dating two months back. No obvious physical findings were noted during general examination. She was diagnosed with an oral ulcer at a local clinic, and received antibiotics. However, the symptoms did not improve; the mass became bigger and painful.

**Diagnosis:**

An incisional biopsy of the oral mass was performed, the immunohistochemistry showed that the tumor cells tested positive for myeloperoxidase, CD4, BCL-2, KI-67. Bone marrow aspiration was negative for malignant cells, and the laboratory test results revealed only monocytosis. Standard bone marrow cytogenetic analysis showed a normal karyotype and leukemia-related fusion gene detection was normal. Therefore, the final diagnosis was intraoral primary GS.

**Interventions:**

The patient was treated with a chemotherapy regimen based on idarubicin and cytarabine arabinoside.

**Outcomes:**

After 2 cycles of idarubicin and cytarabine arabinoside regimen chemotherapy, the patient achieved complete remission. The tumor was barely visible in the left maxillary hard palate. There has been no evidence of disease spread and progression after 1 year of follow-up.

**Conclusions:**

Careful morphological and immunohistochemical analyses, correlating with clinical data are necessary to establish the diagnosis of oral primary GS. Early aggressive systemic chemotherapy can effectively relieve symptoms, significantly reducing primary GS conversion into acute myelocytic leukemia and prolonging overall survival.

## 1 Introduction

Granulocytic sarcoma (GS) is a malignant tumor derived from the bone marrow and located outside the bone marrow. The tumor is also called chloroma, extramedullary myeloid tumor, or myeloid sarcoma (MS), which generally presents with a green color due to the presence of myeloperoxidase (MPO).^[1–2]^ GS usually occurs with acute myelocytic leukemia (AML), myelodysplastic syndrome, myeloproliferative neoplasm or as a recurrence of AML. In majority of conditions, it occurs after the onset of leukemia. When it precedes leukemia without any apparent symptoms, it is termed as the primary or isolated type. It is reported that the ratio of primary GS reaches about 0.85% to 2.2% in patients with AML. It has a high risk of progression to AML within months or years.^[3–5]^ GS can be found in any part of the body but more predominantly in the skin, soft tissues, and lymph nodes. Oral cavity GS rarely occurs, and the clinical characteristics are varied and usually atypical.^[6,7]^ Therefore, it is crucial for oral clinicians to be able to accurately diagnose GS, which ensures prompt treatment to control the disease progression and reach a stable remission period.

Here, a rare case of primary GS is reported, presenting with intraoral involvement, including the mucosa of the left maxillary hard palate, and the adjoining edentulous region. Relevant literature was also reviewed to provide clinicians with additional clarifications on the clinicopathologic manifestation, differential diagnosis, treatment regimens, and prognosis of primary GS of the oral cavity.

## 2 Case report

**Figure 1:**
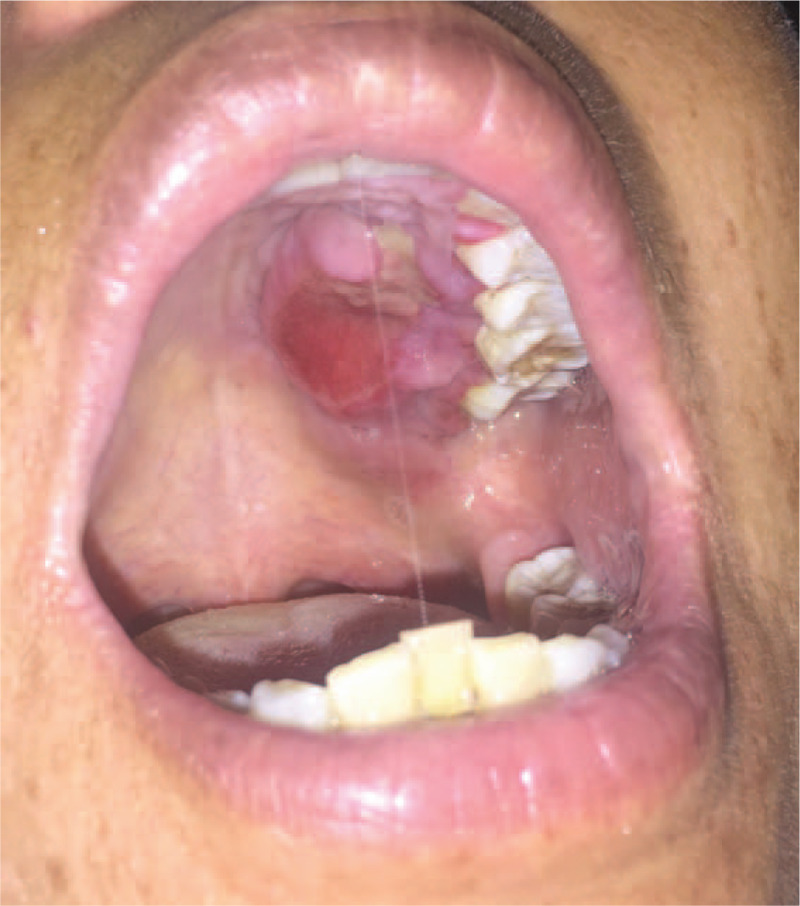
Interoral view a mass in the left maxillary hard palate with ulcerated surface mucosa.

A 49-year-old woman hospitalized at the Department of Plastic and Maxillofacial Surgery, presented with a growing mass in her left maxillary hard palate dating 2 months back. She was diagnosed with an oral ulcer at a local clinic, and received antibiotics. However, the symptoms did not improve; the mass became bigger and painful. Her past medical history was unremarkable without any systemic disease. No obvious physical findings were noted during general examination. She had no bouts of fever, chills, night sweats, vomiting, or weight loss. The intraoral examination revealed that there was a mass measuring 3.0 cm × 2.0 cm in size, with ulcerated surface mucosa in the left maxillary hard palate (Fig. 1).

**Figure 2:**
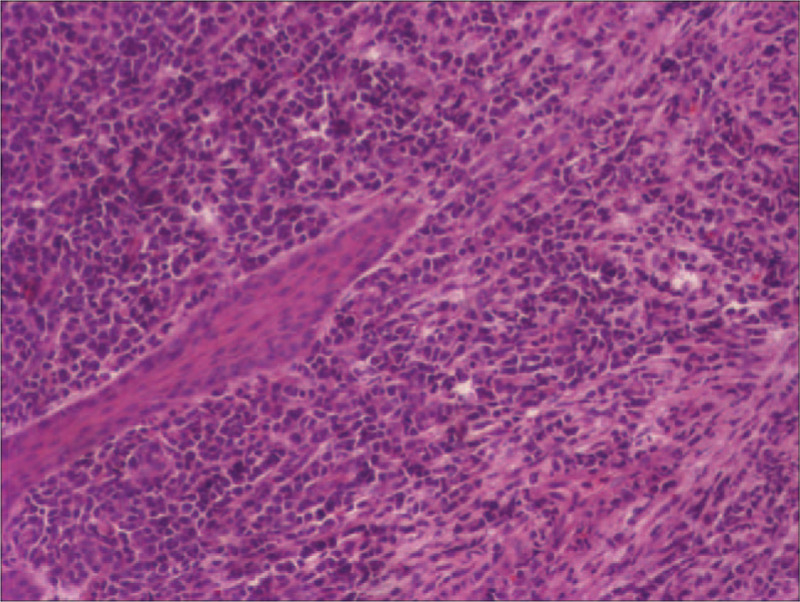
Histologic examination using hematoxylin-eosin (H&E) staining demonstrated that the cell diffuse infiltration growth, most cells were large, the nucleus was vacuolated, the nucleoli were obvious with basophilic cytoplasm containing granules. (magnifification: ×400).

**Figure 3:**
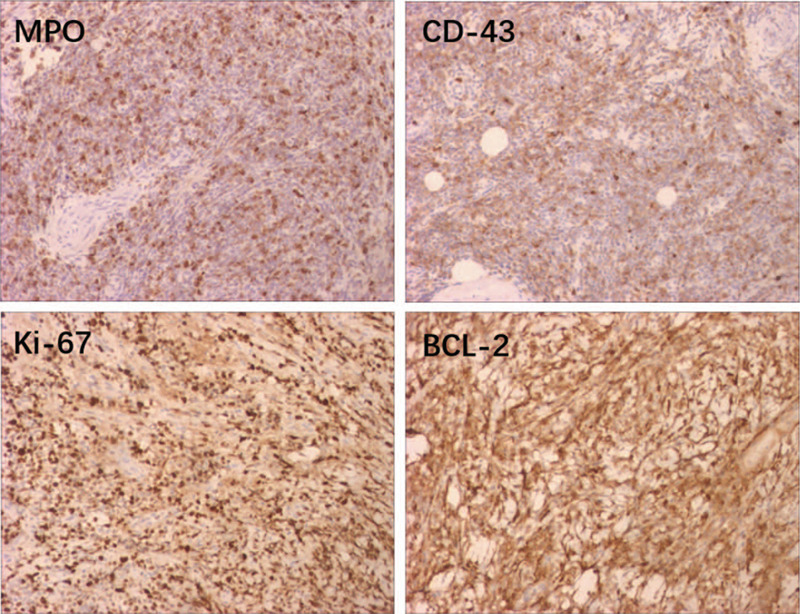
Immunohistochemical staining showed positivity for myeloperoxidase (MPO), BCL-2, CD4, and Ki-67. (magnifification: ×400).

To obtain a definitive diagnosis, an incisional biopsy of the oral mass was performed under local anesthesia. Histologic examination using hematoxylin and eosin staining revealed that there was diffuse cell infiltration growth. Most cells were large, with vacuolated nuclei, obvious nucleoli and a basophilic cytoplasm containing granules (Fig. 2). For the final diagnosis, immunohistochemistry (IHC) was performed, and the tumor cells tested positive for MPO, CD4, BCL-2, KI-67 (Fig. 3), and CD117 and negative for CD3, CD5, CD20, CD56, bcl-6, Mum-1, CD123, MUM-1, TdT, Syn, SOX11, and C-myc. These results supported the diagnosis of GS.

The patient was then referred to the Hematology Department. Bone marrow aspiration was negative for malignant cells, and the laboratory test results revealed only monocytosis. Standard bone marrow cytogenetic analysis showed a normal karyotype and leukemia-related fusion gene detection was normal. Therefore, according to IHC and bone marrow aspiration results in combination with morphological features, the final diagnosis was intraoral primary GS.

**Figure 4:**
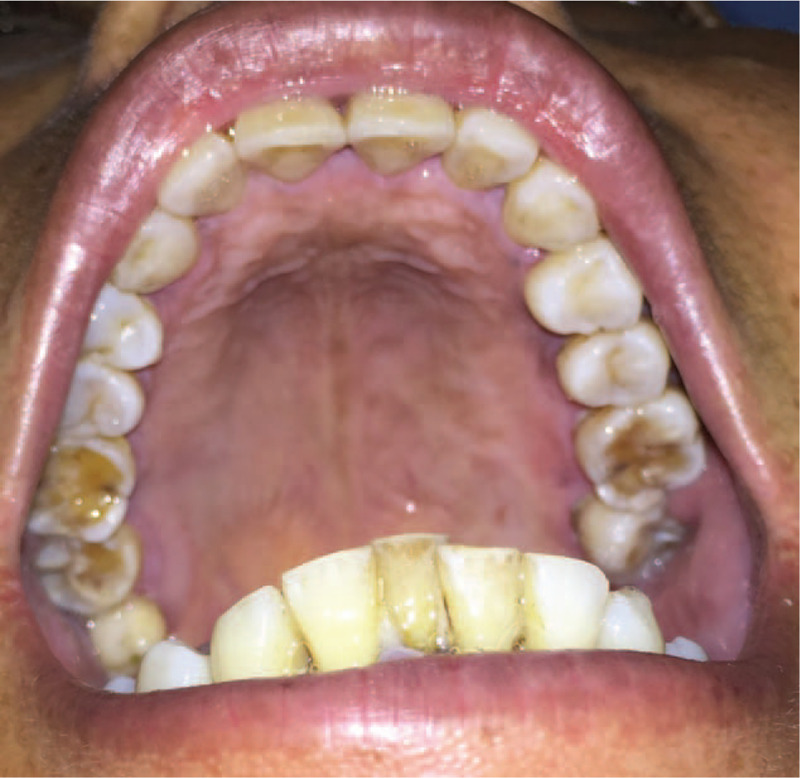
Interoral view after 2 cycles of chemotherapy.

A chemotherapy regimen comprising idarubicin 8 mg VD d_1–3_ and cytarabine arabinoside 150 mg VD d_1–7_ idarubicin and cytarabine arabinoside was initiated. During chemotherapy, the patient only appeared mild nausea, vomiting and canker sores (grade II). These symptoms gradually eased without special therapy. After 2 cycles of idarubicin and cytarabine arabinoside regimen chemotherapy, the patient achieved complete remission. The tumor was barely visible in the left maxillary hard palate (Fig. 4). The patient denied consolidation therapy or bone marrow transplant. Nevertheless, there has been no evidence of disease spread and progression after 1 year of follow-up.

## 3 Discussion

GS is an extramedullary solid tumor composed of immature myeloid cells. First described by Burns in 1811, then in 1853, King named GS as “chloroma,” due to its green colored appearance when exposed to air, resulting from the presence of myeloperoxidase in the tumor cells. In 1966, Rappaport formally proposed the concept of GS. The classification includes non-leukemic GS (isolated or primary GS) and leukemic GS (extramedullary infiltration of leukemia).^[8,9]^

Primary GS occurs in approximately 2 per million persons. The oral cavity occurrence of primary GS is extremely rare. The clinical characteristics of oral primary GS are variable and usually nonspecific.^[10]^ It is a challenge to diagnose oral primary GS based on symptoms and through routine examination. To improve the awareness of the disease, we retrospectively analyzed all cases of oral primary GS, which the tumor precedes leukemia without bone marrow involvement. There are only 31 cases published in PubMed literature together with our case (Table 1).^[11–41]^ The average age of the patients was 44 years (ranging from 2 to 89 years), with a predilection for females (2:1). The tumor was mostly isolated. Commonly involved sites were the gingiva (40.6%), mandible (21.9%), and hard palate (15.6%). Only a few involved the lip, buccal mucosa, and multiple sites. The most common clinical feature of oral primary GS is a painful swelling or a nodule with a reddish to brownish ulcerated surface. In addition, imaging changes that present as soft tissue-occupying lesions, with or without bone erosions, are atypical.^[42–43]^ Therefore, it is usually misdiagnosed as a dental ulcer, epulis and gingival hyperplasia, as well as malignant neoplasms. The challenge to dental practitioners is in differentiating malignant, infectious, and inflammatory lesions, which can often have overlapping clinical features.^[44]^ To accurately diagnose oral primary GS, it is usually based on histopathological and immunohistochemical analysis, and a history of symptoms associated with hematological diseases that might be absent. Morphologically, oral primary GS exhibits variable numbers of primitive, poorly differentiated cells with granular cytoplasm, round to oval nuclei with well-defined membrane and prominent nucleoli, intermingled with reactive inflammatory infiltrate.^[45]^ Different phases of myeloid differentiation are shown in tumor cells containing the eosinophilic myelocytes and blastic cells with minimal granulocytic differentiation. It seems difficult to differentiate the histopathological diagnosis of GS from that of large B-cell lymphoma, Burkitt lymphoma, lymphoblastic lymphoma, and poorly differentiated squamous cell carcinoma. IHC can improve diagnostic accuracy.^[16,25]^ IHC proves the granulocytic origin of tumor cells, which are usually positive for MPO, lysozyme, CD34, CD45, CD68, CD117, and some important markers for the diagnosis of oral primary GS (Table 1). Thereinto, MPO and lysozyme are specific markers of oral primary GS and are associated with the process of tumor cell differentiation.^[3,6,45,46]^

**Table 1:**
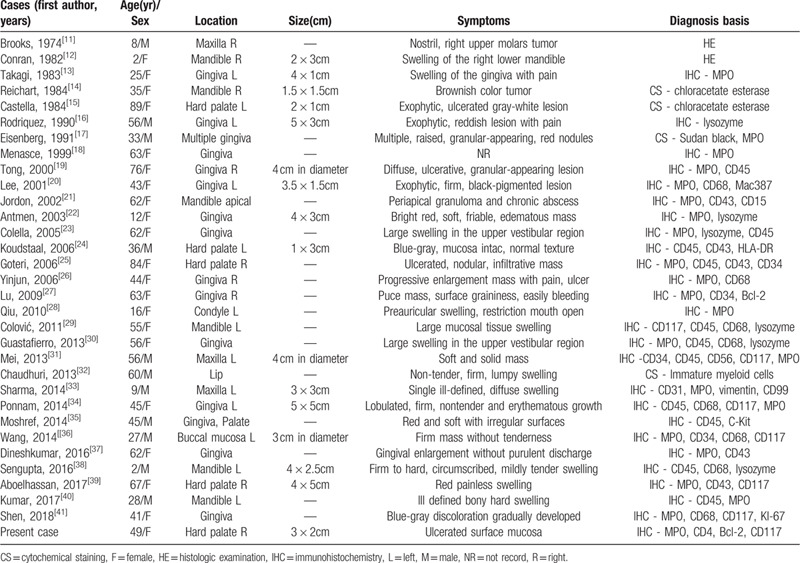
Clinical characteristics and diagnosis of reported cases with primary oral cavity GS.

For nonleukemic GS, it is critical to comprehensively analyze both morphological and immunophenotypic results. In the present case, the pathomorphological examination was nonspecific, but IHC showed positive for EBER, MPO, CD4, BCL-2, CD117, which hinted that tumor cells were from the myeloid cells. IHC was negative for CD3 and CD20. This did not support the source of T and B cells.^[47,48]^ Therefore, the definitive diagnosis was GS. Moreover, before the diagnosis of primary GS, we viewed the bone marrow image, chromosome karyotype analysis, and fusion gene detection. Bone marrow changes occurred before peripheral blood changes, and chromosome karyotype analysis and fusion gene detection could prompt diagnosis when there was no obvious abnormality in bone marrow cell morphology.^[49]^

With regards to available therapeutic options, there is an absence of a treatment guideline for primary GS with the recommended treatment regimen being conventional AML type chemotherapeutic protocols. As Table 2 shows, majority of patients have shown complete remission of the disease after the advent of Ara-c and anthracycline-based therapeutic regimens. It is recommended that early aggressive systemic chemotherapy contributes in the control of the progression of the disease and lengthens survival.^[50,51]^ In some cases, radiotherapy alone can also produce good curative effect, but it cannot delay the transformation of primary GS into leukemia or improve the prognosis.^[11,32]^ Clinically, radiotherapy combined with chemotherapy is an effective choice for clinical symptom relief and consolidation therapy. Surgical resection alone is insufficient.^[31,36,39]^ Whether surgery depends on the size change of the tumor after radiotherapy and chemotherapy is unclear.

**Table 2:**
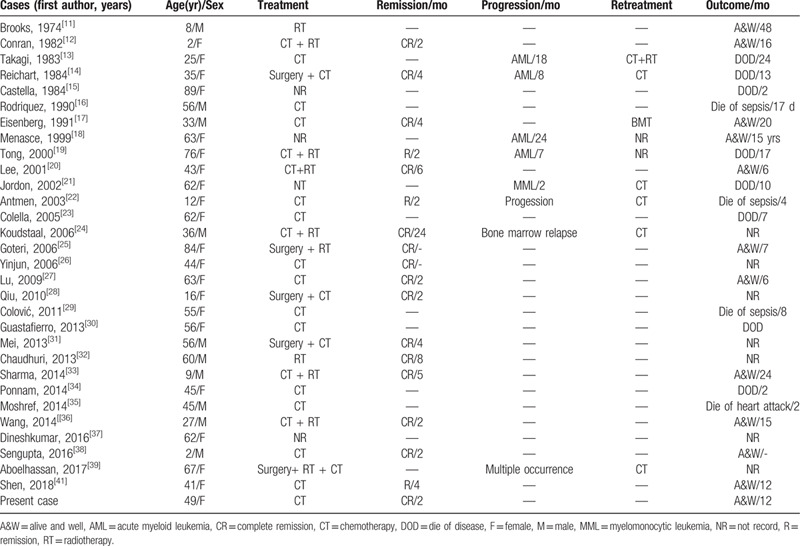
Treatment management and prognosis of reported cases with primary oral cavity GS.

Although systemic chemotherapy can reduce the risk of GS conversion to AML, it cannot completely stop the progression of AML (averagely 10.5 months).^[52]^ At the same time, chemotherapy has side effects such as cardiotoxicity and myelosuppression. Some patients die of sepsis before the end of chemotherapy.^[11,22,29]^ Therefore, choosing a safe and effective treatment method that will prolong long-term survival is difficult. In recent years, with the development of bone marrow transplantation (BMT) and hematopoietic stem cell transplantation (HSCT) technology, the treatment of primary GS has entered the era of comprehensive treatments such as combined radiotherapy and chemotherapy with BMT/HSCT. While there are no prospective trials evaluating the role of BMT/HSCT in primary GS, some retrospective studies have shown good results with a 5-year survival rate of 48% and even encourage considering allogenic BMT/HSCT after the patients’ first induction of remission.^[53–55]^

Targeted therapy is a new orientation of primary GS treatment. These agents include histone deacetylase inhibitors, DNA methyltransferase inhibitors, FLT3 inhibitors, and farnesyl-transferase inhibitors. However, it was most reported by cases lacking multicenter controlled trials.^[50,56]^ With the deepening of the study of primary GS molecular mechanisms, targeted therapy may be a new effective therapy.

## 4 Conclusion

Oral primary GS is a rare tumor with poor clinical outcome. It has protean clinical manifestations and histological overlap with numerous tumors making it a diagnostic challenge for clinicians and pathologists alike. Careful morphological and immunohistochemical analyses, correlating with clinical data are necessary to establish the diagnosis of oral primary GS. Early aggressive systemic chemotherapy can effectively relieve symptoms, significantly reducing primary GS conversion into AML and prolonging overall survival. However, there is no uniform standard on radiotherapy whether combined with systemic chemotherapy synchronously or after the therapy. There is no verdict that BMT/HSCT should be used as preferred alternative for the treatment of disease progression after chemoradiotherapy. Moreover, there is a lack of effective management over long-term treatment. Considering the abovementioned findings, there is a need for further studies to be done.

## Acknowledgments

We would like to thank Editage (www.editage.cn) for English language editing.

## Author contributions

**Conceptualization:** Yun-gang Hu.

**Data curation:** Yun-gang Hu, Xiao-Hua Deng.

**Formal analysis:** Yun-gang Hu.

**Methodology:** Yun-gang Hu, Wei Lei.

**Project administration:** Xiao-lin Li.

**Supervision:** Xiao-lin Li.

**Visualization:** Yun-gang Hu.

**Writing – original draft:** Yun-gang Hu.

**Writing – review & editing:** Xiao-lin Li.

## References

[1] Stoopler Eric T Pinto Andres Alawi Faizan Granulocytic sarcoma: an atypical presentation in the oral cavity. Spec Care Dentist 2004;24:65–9. 15200230 10.1111/j.1754-4505.2004.tb01681.x

[2] Campidelli Cristina Agostinelli Claudio Stitson Richard Myeloid sarcoma: extramedullary manifestation of myeloid disorders. Am J Clin Pathol 2009;132:426–37. 19687319 10.1309/AJCP1ZA7HYZKAZHS

[3] He Jingsong Zhu Lixia Ye Xiujin Clinical characteristics and prognosis of nonleukemic myeloid sarcoma. Am J Med Sci 2014;347:434–8. 23928547 10.1097/MAJ.0b013e31829ca859

[4] Lou Yinjun Qian Wenbin Meng Haitao Frequent extramedullary recurrence of isolated myeloid sarcoma in the long-term follow-up. Ann Hematol 2012;91:1317–9. 22358067 10.1007/s00277-011-1386-x

[5] Vardiman James W The World Health Organization (WHO) classification of tumors of the hematopoietic and lymphoid tissues: an overview with emphasis on the myeloid neoplasms. Chem Biol Interact 2010;184:16–20. 19857474 10.1016/j.cbi.2009.10.009

[6] Yilmaz Asu F Saydam G Sahin F Granulocytic sarcoma: a systematic review. Am J Blood Res 2013;3:265–70. 24396704 PMC3875275

[7] Wilson Carla S Medeiros L Jeffrey Extramedullary manifestations of myeloid neoplasms. Am J Clin Pathol 2015;144:219–39. 26185307 10.1309/AJCPO58YWIBUBESX

[8] Burns A *Observations of Surgical Anatomy, Head and Neck*. Edinburgh, Scotland: Thomas Royce; 1881:364–366.

[9] King A A case of chloroma. Monthly J Med 1853;17:97.

[10] Magdy Mohamed Abdel Karim Nagla Eldessouki Ihab Myeloid sarcoma. Oncol Res Treat 2019;42:224–9. 30840960 10.1159/000497210

[11] Brooks HW Evans AE Glass RM Chloromas of the head and neck in childhood. The initial manifestation of myeloid leukemia in three patients. Arch Otolaryngol 1974;100:306–8. 4528469 10.1001/archotol.1974.00780040316014

[12] Conran MJ Keohane C Kearney PJ Chloroma of the mandible: a problem of diagnosis and management. Acta Paediatr Scand 1982;71:1041–1043. 6961728 10.1111/j.1651-2227.1982.tb09573.x

[13] Takagi M Ishikawa G Kamiyama R Granulocytic sarcoma of the jaw. Bull Tokyo Med Dent Univ 1983;30:1–7. 6572567

[14] Reichart PA von Roemeling R Krech R Mandibular myelosarcoma (chloroma): primary oral manifestation of promyelocytic leukemia. Oral Surg Oral Med Oral Pathol 1984;58:424–7. 6593670 10.1016/0030-4220(84)90337-2

[15] Castella A Davey FR Elbadawi A Granulocytic sarcoma of the hard palate: report of the first case. Hum Pathol 1984;15:1190–1192. 6594316 10.1016/s0046-8177(84)80316-0

[16] Rodriguez JC Arranz JS Forcelledo MF Isolated granulocytic sarcoma: report of a case in the oral cavity. J Oral Maxillofac Surg 1990;48:748–52. 2193130 10.1016/0278-2391(90)90065-a

[17] Eisenberg E Peters ES Krutchkoff DJ Granulocytic sarcoma (chloroma) of the gingiva: report of a case. J Oral Maxillofac Surg 1991;49:1346–50. 1955927 10.1016/0278-2391(91)90317-f

[18] Menasce LP Banerjee SS Beckett E Extra-medullary myeloid tumour (granulocytic sarcoma) is often misdiagnosed: a study of 26 cases. Histopathology 1999;34:391–8. 10231412 10.1046/j.1365-2559.1999.00651.x

[19] Tong AC Lam KY Granulocytic sarcoma presenting as an ulcerative mucogingival lesion: report of a case and review of the literature. J Oral Maxillofac Surg 2000;58:1055–8. 10981989 10.1053/joms.2000.8752

[20] Lee SS Kim HK Choi SC Granulocytic sarcoma occurring in the maxillary gingiva demonstrated by magnetic resonance imaging. Oral Surg Oral Med Oral Pathol Oral Radiol Endod 2001;92:689–93. 11740487 10.1067/moe.2001.118287

[21] Jordan Richard CK Glenn Luther Treseler Patrick A Granulocytic sarcoma: case report with an unusual presentation and review of the literature. J Oral Maxillofac Surg 2002;60:1206–11. 12378502 10.1053/joms.2002.35036

[22] Antmen B Haytac MC Sasmaz I Granulocytic sarcoma of gingiva: an unusual case with aleukemic presentation. J Periodontol 2003;74:1514–9. 14653399 10.1902/jop.2003.74.10.1514

[23] Colella G Tirelli A Capone R Myeloid sarcoma occurring in the maxillary gingiva: a case without leukemic manifestations. Int J Hematol 2005;81:138–41. 15765782 10.1532/ijh97.e0410

[24] Koudstaal MJ van der Wal KGH Lam KH Granulocytic sarcoma (chloroma) of the oral cavity: report of a case and literature review. Oral Oncol Extra 2006;42:70–7.

[25] Goteri G Ascani G Messi M Myeloid sarcoma of the maxillary bone. J Oral Pathol Med 2006;35:254–6. 16519776 10.1111/j.1600-0714.2006.00336.x

[26] Yinjun Lou Jie Jin Zhimei Chen Granulocytic sarcoma of the gingiva with trisomy 21. Am J Hematol 2006;81:79–80. 10.1002/ajh.20469 16369955

[27] Lu Dong-Hui Chen Fei Zhang Qi-Guo Granulocytic sarcoma of oral cavity: report of two cases. Hua Xi Kou Qiang Yi Xue Za Zhi 2009;27:110–2. 19323411

[28] Qiu Ya-ting Yang Chi Zhang Xiao-hu Primary granulocytic sarcoma of the mandibular condyle presenting with the characteristic green color. J Oral Maxillofac Surg 2010;68:2575–9. 20561729 10.1016/j.joms.2009.09.049

[29] Colović Nataša Jurišić Vladimir Terzić Tatjana Alveolar granulocytic sarcoma of the mandible in a patient with HIV. Onkologie 2011;34:55–8. 21346387 10.1159/000317351

[30] Guastafierro Salvatore Falcone Umberto Colella Giuseppe Gingival swelling and pleural effusion: non-leukemic myeloid sarcoma. Eur J Haematol 2013;91:94. 23336824 10.1111/ejh.12077

[31] Mei KD Lin YS Chang SL Myeloid sarcoma of the cheek and the maxillary sinus regions. J Chin Med Assoc 2013;76:235–8. 23557893 10.1016/j.jcma.2012.12.005

[32] Chaudhuri T Paul S Srivastava K Primary granulocytic sarcoma of lip - a rare extramedullary presentation of myeloid leukemia. Indian J Med Paediatr Oncol 2013;34:126–7. 24049304 10.4103/0971-5851.116212 PMC3764733

[33] Sharma A Singh HP Gupta AA Granulocytic sarcoma in non-leukaemic child involving maxillary sinus with long term follow up: A rare case report. Ann Maxillofac Surg 2014;4:90–5. 24987607 10.4103/2231-0746.133078 PMC4073472

[34] Ponnam SR Srivastava G Jampani N A fatal case of rapid gingival enlargement: case report with brief review. J Oral Maxillofac Pathol 2014;18:121–6. 24959052 10.4103/0973-029X.131938 PMC4065429

[35] Moshref M Lotfi A Mashhadi-Abbas F Granulocytic sarcoma (chloroma) presenting as multiple sites in oral cavity: report of a case. Iran J Cancer Prev 2014;7:53–7. 25250149 PMC4142959

[36] Wang CC Chang KP Chang MS Isolated myeloid sarcoma of the buccal region. Br J Hosp Med (Lond) 2014;75:468–9. 25111100 10.12968/hmed.2014.75.8.468

[37] Dineshkumar T Suresh V Ramya R Primary intraoral granulocytic sarcoma: a rare case presenting as generalized gingival enlargement. J Oral Maxillofac Pathol 2016;20:523–6. 27721621 10.4103/0973-029X.190958 PMC5051304

[38] Sengupta M Das I Chatterjee U De novo myeloid sarcoma involving mandible in a child: report of a rare occurrence. J Oral Maxillofac Pathol 2016;20:304–7. 27601827 10.4103/0973-029X.185911 PMC4989565

[39] Aboelhassan R Ali HA Mohammed A Management of hard palatine fistula caused by granulocytic sarcoma: case report. Gulf J Oncolog 2017;1:72–6. 28272007

[40] Kumar P Singh H Khurana N Diagnostic challenges with intraoral myeloid sarcoma: report of two cases & review of world literature. Exp Oncol 2017;39:78–85. 28361861

[41] Shen Y Zhao L Wu Y Multifocal occurrence of intraoral isolated MS in a patient without leukemic presentation: a case report and literature review. Oral Surg Oral Med Oral Pathol Oral Radiol 2018;125:e42–8. 29269257 10.1016/j.oooo.2017.11.013

[42] Claerhout H Van Aelst S Melis C Clinicopathological characteristics of de novo and secondary myeloid sarcoma: a monocentric retrospective study. Eur J Haematol 2018;100:603–12. 29532520 10.1111/ejh.13056

[43] Meyer HJ Pönisch W Schmidt SA Clinical and imaging features of myeloid sarcoma: a German multicenter study. BMC Cancer 2019;19:1150. 31775680 10.1186/s12885-019-6357-y PMC6882227

[44] A practical approach to diagnose soft tissue myeloid sarcoma preceding or coinciding with acute myeloid leukemia. 10.1016/j.anndiagpath.2014.06.001 24969631

[45] Markoc Fatma Bozdogan Nazan Yükrük Fisun Ardic Granulocytic sarcomas: difficulties in diagnosis. Tumori 2010;96:149–53. 20437873 10.1177/030089161009600124

[46] Mourad W Kfoury H Al Husseini H The value of CD34, myeloperoxidase and chloroacetate esterase (Leder) stain in the diagnosis of granulocytic sarcoma. Ann Saudi Med 2001;21:287–91. 17261930 10.5144/0256-4947.2001.287

[47] Amador-Ortiz C Hurley MY Ghahramani GK Use of classic and novel immunohistochemical markers in the diagnosis of cutaneous myeloid sarcoma. J Cutan Pathol 2011;38:945–53. 22050091 10.1111/j.1600-0560.2011.01809.x

[48] Chang CC Eshoa C Kampalath B Immunophenotypic profile of myeloid cells in granulocytic sarcoma by immunohistochemistry. Correlation with blast differentiation in bone marrow. Am J Clin Pathol 2000;114:807–11. 11068557 10.1309/WWW7-DG6X-HC16-D7J2

[49] Alexiev BA Wang W Ning Y Myeloid sarcomas: a histologic, immunohistochemical, and cytogenetic study. Diagn Pathol 2007;2:42. 17974004 10.1186/1746-1596-2-42 PMC2186303

[50] Avni B Koren-Michowitz M Myeloid sarcoma: current approach and therapeutic options. Ther Adv Hematol 2011;2:309–16. 23556098 10.1177/2040620711410774 PMC3573418

[51] Lan TY Lin DT Tien HF Prognostic factors of treatment outcomes in patients with granulocytic sarcoma. Acta Haematol 2009;122:238–46. 19887783 10.1159/000253592

[52] Chargari C Jacob J Bauduceau O Granulocytic sarcoma in a nonleukemic patient: place of radiotherapy and systemic therapies. Case Rep Med 2011;2011:929161. 21629817 10.1155/2011/929161 PMC3099229

[53] Tan D Wong GC Koh LP Successful treatment of primary granulocytic sarcoma by non-myeloablative stem cell transplant. Leuk Lymphoma 2006;47:159–62. 16321843 10.1080/10428190500301140

[54] Chevallier P Labopin M Cornelissen J ALWP of EBMT. Al-logeneic hematopoietic stem cell transplanta-tion for isolated and leukemic myeloid sarco-ma in adults: a report from the Acute Leuke-mia Working Party of the European group for Blood and Marrow Transplantation. Haema-tologica 2011;96:1391–4. 10.3324/haematol.2011.041418 PMC3166114 21685467

[55] Shimizu H Saitoh T Tanaka M Allogeneic hemato-poietic stem cell transplantation for adult AML patients with granulocytic sarcoma. Leukemia 2012;26:2469–73. 22699453 10.1038/leu.2012.156

[56] Almond LM Charalampakis M Ford SJ Myeloid sarcoma: presentation, diagnosis, and treatment. Clin Lymphoma Myeloma Leuk 2017;17:263–7. 28342811 10.1016/j.clml.2017.02.027

